# Capability in Rockwell C Scale Hardness

**DOI:** 10.6028/jres.105.041

**Published:** 2000-08-01

**Authors:** Walter S. Liggett, Samuel R. Low, David J. Pitchure, John Song

**Affiliations:** National Institute of Standards and Technology, Gaithersburg, MD 20899-8980

**Keywords:** calibration, critical to product quality, experimental design, indentation hardness, measurement system comparison, spatial statistics, standard reference material, surface measurement, test method, trend elimination, uncertainty component

## Abstract

A measurement system is capable if it produces measurements with uncertainties small enough for demonstration of compliance with product specifications. To establish the capability of a system for Rock-well C scale hardness, one must assess measurement uncertainty and, when hardness is only an indicator, quantify the relation between hardness and the product property of real interest. The uncertainty involves several components, which we designate as lack of repeatability, lack of reproducibility, machine error, and indenter error. Component-by-component assessment leads to understanding of mechanisms and thus to guidance on system upgrades if these are necessary. Assessment of some components calls only for good-quality test blocks, and assessment of others requires test blocks that NIST issues as Standard Reference Materials (SRMs). The important innovation introduced in this paper is improved handling of the hardness variation across test-block surfaces. In addition to hardness itself, the methods in this paper might be applicable to other local measurement of a surface.

## 1. Introduction

Systems for hardness measurement do not follow exactly a protocol that one might choose. For this reason, there is a difference between the measurement one obtains and the measurement one wishes to obtain. To characterize this difference, one can perform test measurements and from these compute the measurement uncertainty. This paper shows how to do this.

**Fig. 1:**
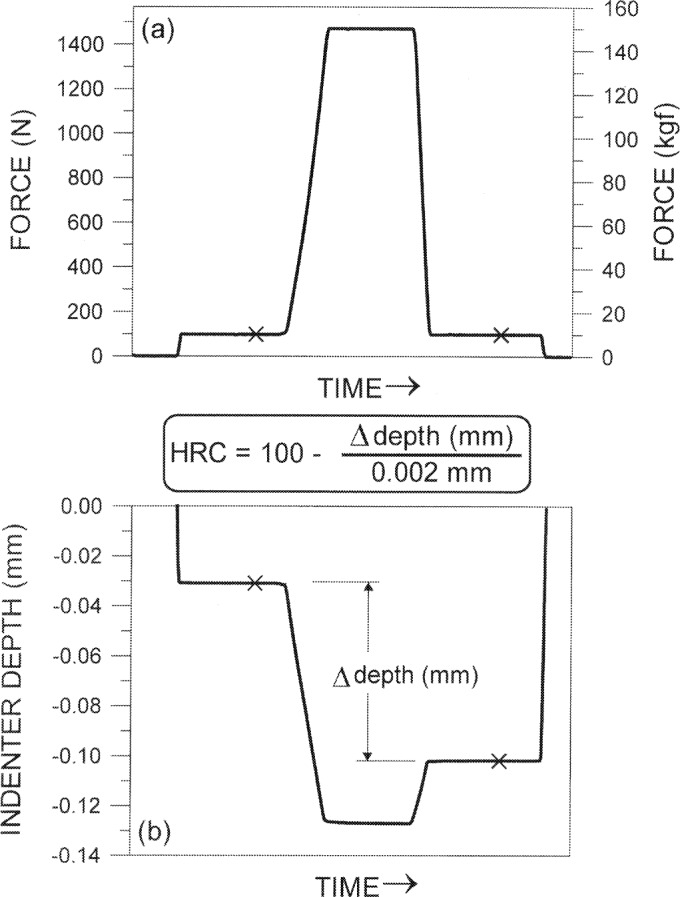
Rockwell C scale hardness test illustrated with change of force with time (a) and resulting indentation depth (b). Indenter depth measurements used in calculation of hardness value are indicated by × symbol.

Rockwell C scale measurement is an indentation test [1,2]. A protocol for performing this test consists of prescriptions for choosing a properly shaped indenter, for driving the indenter into the material, and for calculating the hardness value from the indentation depths. The indenter prescribed is a diamond cone with 120° cone angle blended in a tangential manner with a spherical tip of 200 μm radius. Driving the indenter into the material involves two levels of force, 98.07 N and 1471 N. (These forces can be achieved with masses of 10 kg and 150 kg.) First, as shown in Fig. 1, the smaller force is applied for a prescribed time interval and then the first indentation depth is measured. Second, the force is increased to the higher level in a way that drives the indenter into the material at a prescribed velocity. Third, the larger force is held for a prescribed interval. Fourth, the force is reduced back to the lower level and held for a prescribed interval after which the second indentation depth is measured. Not everyone who chooses a Rockwell C scale protocol chooses the same prescribed intervals and velocity. The American Society for Testing and Materials (ASTM) [1] and the International Organization for Standardization (ISO) [2] only limit the choices allowed. The hardness is calculated from the difference between the second depth and the first. This difference is expressed in multiples of 0.002 mm and subtracted from 100. For steels, Rockwell C scale hardness values are typically between 25 HRC and 65 HRC.

In manufacturing, one generally indexes measurement capability by contrasting measurement uncertainty with the tolerance that the product is required to meet [3]. The tolerance is the difference between the upper and lower specification limits for the product property. In the case of hardness, tolerances are an issue because the bases on which they are set are often obscure. What is usually not made explicit is that hardness is an indicator of a product property critical to quality and that a tolerance on the critical property is the proper basis for a hardness tolerance.

Two features distinguish the approach to uncertainty assessment presented here. The first is reliance entirely on test blocks, and the second is specification of block locations for test measurements. Although we do not deal with it here, one can obtain insight into hardness uncertainty by comparing what one considers to be the ideal protocol with the actual realization of this protocol. Such an approach can be based on propagation of uncertainties [4]. For some deviations of the realization from the ideal, such as a deviation in applied forces, in indenter geometry, or in time intervals of force application, one can (1) characterize the deviation as an uncertainty, (2) obtain sensitivity coefficients that relate such deviations to the resulting hardness measurements, and (3) assess the corresponding component of the measurement uncertainty. The coefficients in the second step cannot be obtained mathematically as in the usual propagation of uncertainties but must be obtained experimentally. Rather than taking this three-step approach, we assess the contribution to the uncertainty of such deviations through use of the NIST Rockwell C scale SRMs [5, 6]. Another possibility that we do not deal with here is arbitrary choice of measurement locations on test blocks. Commonly, guidelines recommend use of the average of five measurements taken somewhere on the test block (Annex H in [4]). The computations associated with this are based on the assumption that the locations were chosen randomly. Rather than considering this possibility, we specify measurement locations and proceed accordingly.

The usefulness of measurement uncertainty for decision making increases when the relation between components of uncertainty and the physical mechanisms that affect the measurement system are clarified. In its organization, this paper proceeds component by component giving for each component the needed test measurements and analysis. Although we do not define a component for each possible mechanism, the components we define can be related to groups of possible mechanisms and further distinctions are sometimes possible. Thinking in terms of mechanisms is especially useful in deciding how one’s hardness equipment might be upgraded. Such thinking is also important when one is trying to understand why the results of two hardness measurements differ. Not all mechanisms and thus not all uncertainty components necessarily contribute to the explanation of the difference between two measurements. Thinking in terms of mechanisms allows one to decide which components can properly be used to explain an observed inconsistency.

The mention of equipment upgrades raises the question of whether the expense is necessary. This involves not only the measurement uncertainty but also the product requirements that the measurement system is to ensure. This issue is the subject of the next section.

The remaining sections explain how to assess experimentally each uncertainty component. The experimental work makes use of test blocks, which introduce another source of variation, the nonuniformity of the hardness across test block surfaces. Dealing with this source of variation as one assesses the hardness uncertainty components is the subject of Secs. 3–5.

As usually applied, Rockwell C scale hardness is a test method that indicates properties of an entire unit through measurement of only a small portion of the unit. Development of test methods with this characteristic is important beyond hardness applications. For example, under the name “combinatorial methods,” there is considerable interest in experiments involving runs under many different conditions but with only a small quantity of material for each condition, a quantity so small that only an indicator of the desired product property can be measured. Because such experiments are usually performed with material samples laid out on a surface, the uncertainty analysis methods in this paper may be instructive.

## 2. Product Specification Limits

Rockwell C scale hardness is used to test the safety of airplane landing gear, the strength of fasteners, the safety of gas cylinders under accidental impact, and the performance of rotary lawn mower blades. These product characteristics and hardness are related in the case of steel because both are affected by heat treatment. Excess heat treatment leads to landing gear that snap, gas cylinders that shatter, and mower blades that fracture. Insufficient heat treatment leads to landing gear that bends, gas cylinders that deform under stress, and mower blades that become dull rapidly.

Clearly, some product characteristics measured by Rockwell C scale hardness are critical to quality. Manufacturers of products for which this is true have little choice but to develop and maintain appropriate capability in hardness measurement. An alternative test method is, of course, a possibility, but such development would likely be very expensive. Thus, treating Rockwell hardness with disdain because of its simple protocol and long history is not an option.

For some product characteristics but not hardness, the engineering knowledge needed to determine acceptable values of the characteristic, that is, to set specification limits, is available without further experiment. For example, say that the characteristic is the shape of a part. To calculate specification limits, one could envision how deviations from the ideal shape would affect the use of the part, identify various part dimensions that together can be employed to assure successful usage, and finally specify limits on these part dimensions. Similarly, say that the characteristic is the composition of some material. To calculate specification limits, one could anticipate how various constituents would affect usage and set specification limits on the concentrations of these constituents. These are examples of product characteristics for which one can set specification limits theoretically.

The case of Rockwell C scale hardness is different. In fact, this difference is sometimes emphasized by saying that determinations of dimension and of concentration are measurements whereas Rockwell hardness is a test method. As illustrated by Rockwell hardness, test-method protocols are sometimes quite removed from product characteristics of real interest. There is no quantitative model that connects Rockwell hardness and the performance of airplane landing gear under stress. The only way to establish the connection is through experiment. In the case of landing gear, this effort involves determination of the relation between tensile strength and hardness. In fact, one can find publications that give this relation [7], but one must be concerned with the reliability of the published relation and whether it holds for all classes of steel. It would seem that in many cases, the use of a test method to assure a product characteristic requires costly experimental work and that one would only do this work if the characteristic were critical to quality and a test method were the only way to gauge the characteristic.

To compensate for problems in the experimental work connecting product characteristic and test method, one might think of tightening the specification limits until one is sure that test method results indicate satisfactory product performance. This reasoning may be behind some customer-supplier agreements that contain specification limits on Rockwell C scale hardness. Tightening specification limits, of course, results in more stringent requirements on the uncertainty of the test method. Reducing this uncertainty may be difficult. In this case, one must decide what experimental work to invest in: One could improve one’s understanding of the specification limits or reduce one’s uncertainty in use of the test method.

The idea that specification limits drive uncertainty requirements is complicated in the case of Rockwell C scale hardness because of differences among uncertainty components. The most important difference is between the components that describe the variation of a particular testing system and the components that arise when one is comparing systems. Characterization of the former entails what is commonly referred to as gage repeatability and reproducibility (gage R&R) [3]. Characterization of the latter involves comparison of testing machines and indenters.

Rockwell C scale measurement can be used to reduce the variation in a heat treatment process. Although reduced variation alone does not guarantee that the parts produced will be satisfactory, such reduction is a reasonable first step. If this is one’s goal, one should use only a single hardness measurement system. The measurement capability needed involves only the short and long term variation of this system. Thus, a gage R&R study is sufficient to characterize the uncertainty components of interest and thus to determine whether the hardness system is capable for the task.

More broadly, one must consider the relation of one’s hardness measurements to the hardness measurements that were part of setting the specification limits. Generally, these two sets of measurements will involve different machines and indenters. Despite the differences, the specification limits must apply to measurements from both. There might be just two systems involved, the supplier’s and the customer’s. Alternatively, both systems might be referred to the hardness measurements made by NIST. In either case, the uncertainty components that characterize the machine and indenter errors enter the determination of measurement capability. Often, these errors are larger than the errors apparent in a gage R&R study, and determining how to reduce them by upgrading a measurement system is more difficult.

## 3. Gage R&R

### 3.1 Assessment With Nonuniform Blocks

Repeatability, the first “R” in “R&R”, is defined as “closeness of the agreement between the results of successive measurements of the same measurand carried out under the same conditions of measurement” [4]. Two stipulations in this definition, same measurand and same conditions, require care. Successive measurements of the same measurand cannot be realized because a hardness measurement can be made only once at each test block location and test block hardness is not perfectly uniform. Thus, a way around block nonuniformity must be employed. In the definition, the words “same conditions” must be augmented by spelling out the conditions to be held constant.

For the dead-weight, Rockwell-hardness machine at the NIST [6], we specify that “successive measurements…under the same conditions” means an uninterrupted sequence of measurements made with the same indenter and machine configuration. Our use of the word “uninterrupted” is intended to imply that the measurements are made one after another over a short period of time. “Made with the same machine configuration” means made without changing the test cycle sketched in Fig. 1 or changing the setting of the machine in any other way. Because the NIST machine has a flat anvil, we add the proviso that in the course of the measurements, the block not be removed from the anvil. For testing machines of other designs, other provisos might be suitable. Subsequently in this paper, we frequently stipulate that measurement conditions be held constant as in repeatability assessment.

We define repeatability in terms of measurements on a perfectly uniform test block and then show how repeatability can be estimated with nonuniform test blocks. The key is prescription of measurement locations on the test blocks. The prescribed locations must be far enough apart to avoid the hardening caused by the residual stress left by an indentation and must be symmetrical and close enough that deviations due to block nonuniformity cancel. Most of the methods presented in this section are based on measurement patterns composed of hexagons with 6 mm between adjacent vertices. The distance 6 mm seems large enough to avoid the crowding effect and small enough to allow sufficient indents on a block. Measuring at the vertices and center of a 6 mm hexagon requires that one develop one’s experimental technique. This can be done with some effort.

**Fig. 2:**
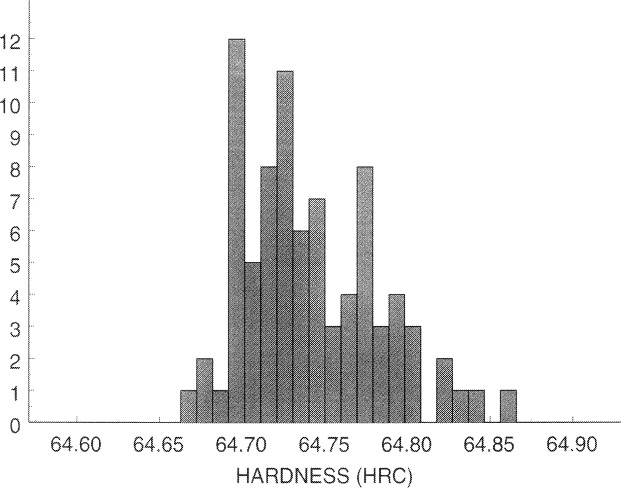
Histogram of measurements taken on block 95N63001.

**Fig. 3:**
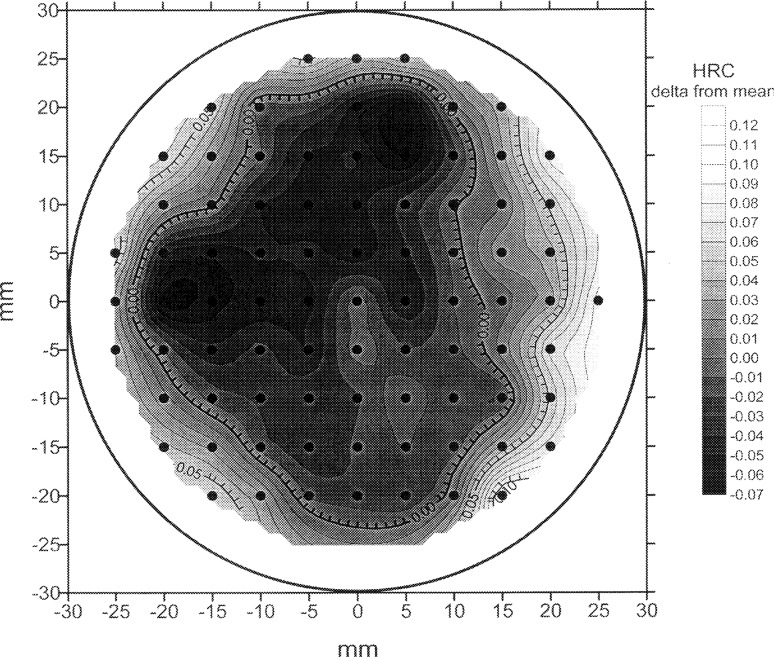
Contour plot for block 95N63001 obtained with commercial software.

That block nonuniformity cannot be ignored when the machine has good repeatability is illustrated by the measurements that are presented in Fig. 2 as a histogram. NIST obtained these 83 values by measuring one of the SRM blocks, 95N63001, on a square 5 mm grid. The measurement spread shown in this histogram is caused by both lack of repeatability and block nonuniformity. These two sources can be distinguished if the location of the measurements is taken into account. Block nonuniformity is largely characterized by smooth variation with location because the major causes of this nonuniformity, variation in the material and the heat treatment applied to it, vary smoothly. Thus, the hardness contour plot in Fig. 3 indicates how much the block nonuniformity contributes to the measurement spread shown in Fig. 2. Figure 3 shows that this block is harder near its edges and softer in the middle. Moreover, the variation shown by the contours largely explains the spread in Fig. 2. Not only do Figs. 2 and 3 illustrate the possible impact of block nonuniformity on uncertainty assessment, they also illustrate that the basis for dealing with block nonuniformity is the smooth variation of hardness with location.

### 3.2 Trend Elimination

**Fig. 4:**
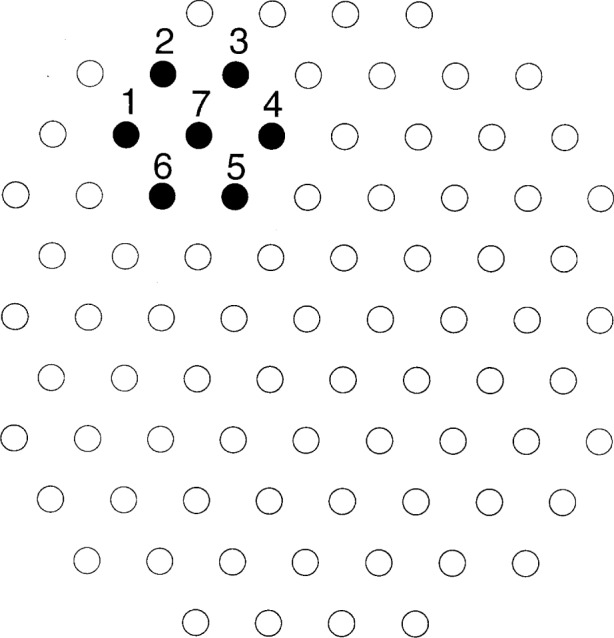
A 6 mm equilateral triangular grid for a block with a hexagonal measurement pattern highlighted and numbered.

As the basis for the methods in this section, we assume that hardness variation over a 6 mm hexagon resembles variation with a constant gradient. (See Sec 3.8 for further discussion of this approximation.) Figure 4 shows a 6 mm hexagon with numbered vertices that is embedded in an equilateral triangular grid covering a block 52 mm in diameter. Say that we measure the center point, number 7, and a cross-hexagon pair, perhaps vertices 1 and 4, holding measurement conditions constant as in repeatability assessment. If the hardness has a constant gradient, then, except for the variation due to lack of repeatability, the center measurement equals the average of the other two measurements. By examining the deviation from equality, we can assess the repeatability without concern for block nonuniformity. In addition, arrangement of measurements on a 6 mm hexagon can be used to eliminate block nonuniformity in system comparisons such as comparison of indenters. If we choose conditions for the center measurement that differ from those for the other two, then we can determine the effect of this difference hindered only by the lack of repeatability, not by block nonuniformity.

The foregoing can be expressed mathematically. As shown in Fig. 4, let the locations on the outside of the hexagon be numbered clockwise from 1 to 6, and let the center be numbered 7. The 3 cross-hexagon pairs are vertices 1 and 4, vertices 2 and 5, and vertices 3 and 6, respectively. The *x-y* coordinates of the locations are approximately given in millimeters by (−6,0), (−3,5), (3,5), (6,0), (3, −5), (−3, −5), and (0,0). Let the seven hardness measurements be denoted *H_i_*, *i* = 1,…,7. Let each cross-hexagon pair be measured under constant conditions as in repeatability assessment. The comparisons are among (*H_i_* + *H_i_*_+3_)/2, *i* = 1,…,3 and *H*_7_. If the block nonuniformity has a constant gradient, then the actual hardness as a function of *x–y* coordinates on the surface is given by *b*_0_ + *b*_1_ x*_i_* + *b*_2_ *y_i_* for some coefficients *b*_0_, *b*_1_, and *b*_2_. We see that nonuniformity with this dependence on location does not enter the comparisons.

It is useful to express the hardness measurements in a way that makes explicit the effect of the lack of repeatability. Consider the general case of system comparisons. In this case, each cross-hexagon pair is measured under the same conditions, but different pairs and the center point may be measured under different conditions. We denote the system conditions for vertices 1 and 4 by A, the conditions for 2 and 5 by B, the conditions for 3 and 6 by C, and the conditions for the center point by D. We have

$$\begin{array}{c} \left(H_{1}+H_{4}\right)/2=\beta_{A}+\left(\varepsilon_{1}+\varepsilon_{4}\right)/2\\ \left(H_{2}+H_{5}\right)/2=\beta_{B}+\left(\varepsilon_{2}+\varepsilon_{5}\right)/2\\ \left(H_{3}+H_{6}\right)/2=\beta_{C}+\left(\varepsilon_{3}+\varepsilon_{6}\right)/2\\ H_{7}=\beta_{D}+\varepsilon_{7} \end{array}$$

The *ε*_i_, *i* = 1,…,7, denote the effects of the lack of repeatability. We model these seven effects as statistically independent with zero mean and standard deviation *σ*. We can think of *β*_A_, *β*_B_, *β*_C_, *β*_D_ as the hardness values one would obtain at the center point of the hexagon under the four sets of conditions and in the absence of any effect of lack of repeatability. If the sets of conditions were the same, these values would be the same.

If we knew the value of *σ*, then we could obtain a confidence interval for *β*_A_ – *β*_B_, say. The standard deviation of two-location averages is $\sigma /\sqrt{2}$. Recall that the variance (the square of the standard deviation) of a sum of independent errors is given by the sum of the variances of the individual errors. A 95 % confidence interval for *β*_A_ – *β*_B_ is given by

$$\left(H_{1}+H_{4}\right)/2-\left(H_{2}+H_{5}\right)/2\pm 1.96\;\sigma .$$

The formula that applies when the center value is involved is somewhat different. A 95 % confidence interval for *β*_A_ – *β*_D_, say, is given by

$$\left(H_{1}+H_{4}\right)/2-H_{7}\pm 1.96\;\sqrt{3/2\;}\sigma .$$

Other comparisons are analogous to one or the other of these formulas. Note that whether or not these comparisons are satisfactory for the purpose depends on the size of the standard deviation *σ*. If the standard deviation is too large, more precise comparisons can be made by improving the repeatability of the machine or by repeating the measurements on hexagons laid out elsewhere on the block.

### 3.3 Assessing Repeatability

Consider measurement of all seven points of a 6 mm hexagon under constant conditions as in repeatability assessment. As explained in the previous section, if the block nonuniformity has constant gradient, only the lack of repeatability causes (*H*_1_ + *H*_4_)/2, (*H*_2_ + *H*_5_)/2, (*H*_3_ + *H*_6_)/2, and *H*_7_ to differ. Moreover, only lack of repeatability causes *H*_1_ + *H*_3_ + *H*_5_ − *H*_2_ − *H*_4_ − *H*_6_ to differ from 0. Thus, one can estimate *σ*, that is, assess the repeatability, from these quantities.

Usually, one estimates the variance *σ*^2^ and denotes the estimate by *s*^2^. Let the mean of the seven readings be

$$\bar{H}=\frac{1}{7}\sum_{i=1}^{7}H_{i}.$$

The variance estimate is given by

$$s^{2}=\frac{1}{4}[{\left[H_{7}-\bar{H}\right]}^{2}+\sum_{i=1}^{3}2{\left[\left(H_{i}+H_{i+3}\right)/2-\bar{H}\right]}^{2}+{\left[H_{1}+H_{3}+H_{5}-H_{2}-H_{4}-H_{6}\right]}^{2}/6].$$

Because this estimate has only 4 degrees of freedom, its variability must be taken into account when it is used to draw conclusions. If the corresponding standard deviation *s* were to be used instead of *σ* to form confidence intervals, then the appropriate value from the Student’s *t*-table, 2.776, would replace the 1.96 shown above.

For purposes such as reporting the lack of repeatability of a testing machine, one would want an estimate of *σ* with more degrees of freedom. One way to do this is to measure more than one hexagon with the same indenter, say *m* hexagons. Let the hardness measurement for location *i* on hexagon *j* be given by *H_ji_*. Proceeding as above for each hexagon, we obtain an estimate of *σ* for each hexagon, which we denote by *s_j_*. The overall estimate of the standard deviation is given by

$$\sqrt{\frac{1}{4m}\sum_{j=1}^{m}4\;s_{j}^{2}.}$$

This estimate has 4*m* degrees of freedom. More generally, if the estimate *s_j_* had *ν_j_* degrees of freedom, then the overall estimate would have

$$\sum_{j=1}^{m}v_{j}$$

degrees of freedom and would be given by

$$\sqrt{\sum_{j=1}^{m}v_{j}s_{j}^{2}/\sum_{j=1}^{m}v_{j}.}$$

In many cases, the 12 degrees of freedom obtained from 3 hexagons is sufficient.

**Fig. 5:**
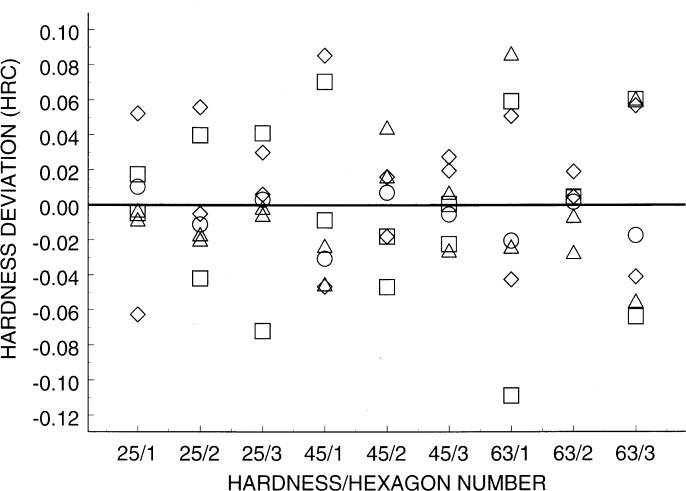
Repeatability data with different symbols for the center point (circle) and each cross-hexagon pair (triangle, square, or diamond).

Figure 5 shows, for each of three hardness levels, the measurement results on three hexagons given as deviations from ${\bar{H}}_{j}$. The units are hardness on the Rockwell C scale (HRC). The center is plotted with a circle, and members of different cross-hexagon pairs are plotted with triangles, squares, and diamonds, respectively. The variation shown is due to block nonuniformity and lack of repeatability. Nonuniformity with a steep gradient would lead to some pairs exhibiting large deviations in opposite directions from the center line. There is evidence of this. Members of pairs do not lie equal distances from the center line because of lack of repeatability.

For the three HRC levels 25, 45, and 63 the standard deviation estimates *s* for the variation due to lack of repeatability are 0.029 HRC, 0.033 HRC, and 0.024 HRC, respectively. Each of these estimates has 12 degrees of freedom. These estimates do not provide definitive evidence that the repeatability varies with hardness level.

### 3.4 Long-Term Variation

Comparison of cross-hexagon averages with center measurements obtained previously is one way to monitor the long-term variation of a hardness measurement system. One begins by measuring the centers of enough hexagons to cover the period during which monitoring is to occur. This may require laying out hexagons on several blocks of the same hardness level. One measures the centers under the constant conditions necessary to avoid any variation beyond the inevitable lack of repeatability. Then, over the monitoring period, one measures cross-hexagon pairs and compares pair averages with the center. Note that this approach cancels both block nonuniformity and block-to-block variation. For this reason, we can combine readings from hexagons on different blocks in the same way we combine readings from hexagons on the same block.

To describe this approach mathematically, let the outer points of each hexagon be indexed by *i* as above, and let *j* index the hexagons. Denote the center measurement for hexagon *j* by *H*_0_*_j_*. The order in which the cross-hexagon pairs are measured during the monitoring is important. It is perhaps best to measure one pair on each hexagon before returning to measure a second pair on any hexagon. Thus, for some ordering of the hexagons, one measures a pair on each hexagon, then a second on each, and finally a third. For each monitoring point, one can place on a run chart the deviation of the across-hexagon average from the center-point measurement

$$\left(H_{ji}+H_{j\left(i+3\right)}\right)/2-H_{0j.}$$

Frequency of monitoring and the measurement conditions to be held constant during monitoring are both issues to be decided.

**Fig. 6:**
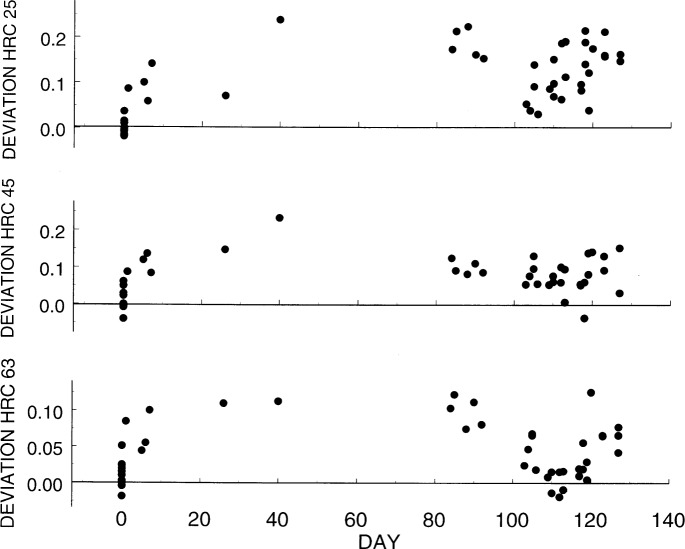
Long-term variation shown for each day by the difference between a cross-hexagon pair and the center.

For each hardness level, Fig. 6 shows a run chart with these deviations plotted versus time. On the day the center measurements were made, three hexagons for each level were measured fully to assess the repeatability. These measurements are the ones shown in Fig. 5. From these data, we computed the nine deviations shown at day 0. A monitoring deviation was obtained each day the machine was to be used, but as shown, the machine was used irregularly during the period portrayed. On some days, two or even three monitoring deviations were obtained. All of these are shown in Fig. 6.

The run charts in Fig. 6 are worrisome because there seem to be special causes that cannot be easily identified. Clearly, the machine was operating differently on the day that the center measurements were made. For this reason, almost all the deviations are positive. Moreover, during the period covered by the last part of the chart, the deviations seem to fall and then recover. Concern about the causes of these appearances is reenforced by the fact that they appear to some degree at each hardness level, although they are most pronounced at the lowest level. The first place one might look for causes is in the mechanical operation of the testing machine. One would like to institute some procedure for using the machine that would assure that whatever the cause, its effect would be limited. Some such procedures, for example, warm up procedures, are already in place but may not be fully effective. In particular, the NIST machine provides a trace of indenter depth versus time for each measurement. At the beginning of each day, this trace is used to detect the need to adjust the moving parts of the machine so that the forces are applied at the proper rates. After some thought, we have concluded that although Fig. 6 is worrisome, the effects are not pronounced enough to permit identification of the causes.

### 3.5 Assessing Reproducibility

Parallel to the definition of repeatability, reproducibility, the second “R” in “R&R”, is defined as “closeness of the agreement between the results of successive measurements of the same measurand carried out under changed conditions of measurement” [4]. What conditions change must be stated. In the operation of the NIST dead-weight Rockwell-hardness machine, the change of primary interest is the change from day to day that occurs with the same indenter and without changing the test cycle sketched in Fig. 1 or resetting the machine parameters in any other way. Often, the changed conditions in discussions of reproducibility involve changes in operator although this is less important with computer-controlled machines. There are also other possibilities for defining changed conditions.

When one thinks of reproducibility, one usually thinks of sources of error in addition to the ones that cause lack of repeatability. Thus, one decomposes a hardness measurement *H* into three terms: *ε*, the term that reflects the error sources that cause lack of repeatability; δ, the term that reflects the additional error sources associated with the lack of reproducibility; and *μ*, the actual hardness biased by the machine and indenter error. We have

H=μ+δ+ε.

In thinking about a measurement system over time, one has to note when each term in this equation changes. In the case of our model of the NIST machine, the term *ε* changes with every new measurement; the term *δ* changes with each new day; and the term *μ* changes only when the machine or indenter is changed intentionally.

The effect of the additional sources of error is to add a term to the cross-hexagon pair (*H_ji_* + *H*_*j*(*i* + 3)_)/2, which we denote *δ_ji_*, and to add a term to the center measurement *H*_0_*_j_*, which we denote *δ*_0_. Say that we use just one cross-hexagon pair for each monitoring point, which we assume involves changes in the additional error sources. Denoting the deviations by *D_ji_*, we have

$$D_{ji}=\left(H_{ji}+H_{j\left(i+3\right)}\right)/2-H_{0j}=\delta_{ji}-\delta_{0}+\left(\varepsilon_{ji}+\varepsilon_{j(i+3)}\right)/2-\varepsilon_{0j}.$$

On the right side of this equation, the error terms are statistically independent if their subscripts differ. We see that the run chart has a constant offset because *δ*_0_ never changes. Moreover, there is some dependence between *D_ji_* and *D_jk_* because *ε*_0_*_j_* only changes with *j*.

Typically, reproducibility is assessed through the use of a standard deviation estimate. This implies that the changes incorporated in the definition of reproducibility must have effects that are reasonably portrayed as random with constant standard deviation. Think of what a run chart fashioned as those discussed above would look like if this were true. Say that the conditions that change in accordance with the reproducibility definition change with every new point on the chart. Then, with one minor departure, the deviations portrayed by the run chart would appear to vary randomly without the amount of variation changing appreciably. The departure is the dependence discussed above caused by three run chart points having a common center measurement. As an aside, note that one could form a run chart with changed conditions only every second point or every third point. Such an alternative is reasonable but requires a somewhat different reproducibility assessment.

If the lack of reproducibility is reasonably summarized by a standard deviation, then we can add control limits to the run chart of the deviations. Say that we have *n* deviations available, which for generality, we take to have been obtained from one or more cross-hexagon pairs on several hexagons. The control limits should be centered at

$$\bar{D}=\frac{1}{n}\sum_{j}\sum_{i}D_{ji},$$

where the sums cover the pairs from various hexagons for which deviations are available. Using 3 times the standard deviation, we obtain for the control limits

$$\bar{D}\pm 3\sqrt{\frac{1}{n-1}\sum_{j}\sum_{i}{\left(D_{ji}-\bar{D}\right)}^{2}.}$$

These control limits account for the repeatability and the reproducibility of the measurement system. Points outside the control limits would indicate a special cause of variation to be investigated. Note that the control limits do not have to be re-estimated even though the monitoring involves hexagons from several blocks.

We now have a model for hardness measurements with two sources of uncertainty, *ε* and *δ*. The variance of *ε* is denoted by *σ*^2^ and is estimated by *s*^2^ as given in Sec. 3.3. The variance of *δ* is denoted by $\sigma_{\delta }^{2}$. An estimate of $\sigma_{\delta }^{2}$ can be obtained from the deviations *D_ji_*. We have

$$s_{\delta }^{2}=\frac{1}{n-1}\sum_{j}\sum_{i}{\left(D_{ji}-\bar{D}\right)}^{2}-\frac{3}{2}s^{2}$$

Note the subtraction of 3*s*^2^/2 to remove from the variability observed on the control chart the part attributable to the lack of repeatability. The stability of this estimate might be seriously compromised if from deviation to deviation, measurement conditions do not change as envisioned in the definition of reproducibility. Note in particular that if the change envisioned is the change from day to day, then measuring several cross-hexagon pairs within a day will not do much to provide a better estimate of $\sigma_{\delta }^{2}$.

The desirability of randomness in the sequence of deviations *D_ji_* arises from the idea that randomness implies that each deviation is the effect of many causes none of which is dominant. If there are many causes, then one expects the statistical properties of the deviations, their mean and standard deviation, to remain constant in the future. What is worrisome about Fig. 6 is that there is some indication that a few dominant causes exist that might lead to deviations in the future that are unlike anything observed in Fig. 6.

Is a standard deviation an appropriate summary for Fig. 6? We computed *s_δ_* using only one value of *D_ji_* from days during which two or three were measured. We obtained for the lowest level 0.056 HRC, for the middle level 0.038 HRC, and for the highest level 0.036 HRC. This standard deviation does gauge the day-to-day variation, the error component called lack of reproducibility, observed in Fig. 6. This component seems to be largely the effect of day-to-day variations in the way the machine moves in executing the test cycle. What is worrisome is that *s_δ_* may be too small to cover this error component as it might appear in future measurements. Another caution is that these results might be misleading in the interpretation of combinations of measurements made over successive days because Fig. 6 seems to indicate some lack of randomness, that is, some day-to-day dependence.

### 3.6 Comparison and Assessment

Consider now the comparison of indenters and testing machines. Generally, the notion that changes in indenters or machines are changes that should be incorporated in a definition of reproducibility seems contrived because one usually has only a few indenters or machines with which to experiment. Thus, a statement of differences would seem to be more satisfying than a standard deviation as a way of summarizing the variation observed when the indenter or machine is changed.

The comparisons we consider are set up so that only the lack of repeatability enters. One could do comparisons as suggested in Sec. 3.2 and use the repeatability assessment in Sec. 3.3 to obtain confidence intervals. However, for efficiency, one might compare indenters or machines and assess the repeatability in the same experiment. Say one has 3 indenters to be compared using the 14 locations available in 2 hexagons. Let the indenters be denoted A, B, and C. In each hexagon, we assign indenter A to vertices 1 and 4, B to 2 and 5, and C to 3 and 6. Further, we assign A to the center of hexagon 1 and B to the center of hexagon 2. As above, we denote the hardness reading for location *i* and hexagon *j* by *H_ji_*.

Analysis of this experiment should be done with a program for multiple regression, which most readers will find available on their computers. A multiple regression program models the observations in terms of parameters and errors. In general, the model is given by

y=Xβ+ε,

where ***y*** is the vector of observations, ***X*** is referred to as the design matrix, ***β*** is the vector of parameters, and **ε** is the vector of unknown errors. These errors are due to lack of repeatability in the case considered here. A multiple regression program requires input of ***y*** and ***X***; it returns an estimate of ***β***, which we denote by $\hat{\beta }$.

One might expect that we would take the 14 hardness measurements *H_ji_*; *j* = 1,2; *i* = 1,…,7; as the elements of ***y***, but this leads to the need for elements in ***β*** that model the block nonuniformity across the hexagons. Instead, we have decided to confine the elements of ***β*** to *β*_A_, *β*_B_, ***β***_C_, the hardness readings with indenters A, B, and C, respectively, that one would obtain under perfect repeatability at the center of hexagon 1, and ***Δ***, the difference in hardness between the centers of hexagons 1 and 2. We have

$$\beta =\left(\begin{array}{c} \beta_{A}\\ \beta_{B}\\ \beta_{C}\\ \Delta \end{array}\right).$$

So that we can confine ***β*** to these 4 parameters, we must adopt as elements of **y** the combinations of hardness readings that we introduced in Sec. 3.2. We take as observations the across-hexagon averages and the center reading scaled so that their standard deviation is *σ*. We add $\left(H_{11}+H_{13}+H_{15}-H_{12}-H_{14}-H_{16}\right)/\sqrt{6}$ and the corresponding quantity from the other hexagon so that the regression routine includes them in the estimation of *σ*. The data values to which the regression model is fit are given by

$$y=\left(\begin{array}{c} \left(H_{11}+H_{14}\right)/\sqrt{2}\\ \left(H_{12}+H_{15}\right)/\sqrt{2}\\ \left(H_{13}+H_{16}\right)/\sqrt{2}\\ H_{17}\\ \left(H_{11}+H_{13}+H_{15}-H_{12}-H_{14}-H_{16}\right)/\sqrt{6}\\ \left(H_{21}+H_{24}\right)/\sqrt{2}\\ \left(H_{22}+H_{25}\right)/\sqrt{2}\\ \left(H_{23}+H_{26}\right)/\sqrt{2}\\ H_{27}\\ \left(H_{21}+H_{23}+H_{25}-H_{22}-H_{24}-H_{26}\right)/\sqrt{6} \end{array}\right)$$

As a consequence of our choice of ***β*** and **y**, the design matrix is given by

$$X=\left(\begin{array}{cccc} \sqrt{2} & 0 & 0 & 0\\ 0 & \sqrt{2} & 0 & 0\\ 0 & 0 & \sqrt{2} & 0\\ 1 & 0 & 0 & 0\\ 0 & 0 & 0 & 0\\ \sqrt{2} & 0 & 0 & \sqrt{2}\\ 0 & \sqrt{2} & 0 & \sqrt{2}\\ 0 & 0 & \sqrt{2} & \sqrt{2}\\ 0 & 1 & 0 & 1\\ 0 & 0 & 0 & 0 \end{array}\right).$$

The logic of the model we have adopted can be seen by considering the equation **y** = ***Xβ***, the model for ***y*** with error term removed. For example, from the sixth row, we have

$$\left(H_{21}+H_{24}\right)/\sqrt{2}=\sqrt{2}\beta_{A}+\sqrt{2}\Delta .$$

This shows that the first cross-hexagon average for hexagon 2 is given by the hardness reading obtained from indenter A in hexagon 1 plus the hardness difference between hexagon 1 and hexagon 2. This illustrates the parameterization that we have chosen.

To estimate the elements of ***β***, we need a regression program that runs without adding a constant term to the model. (Adding a constant term consists of adding a column with elements 1 to ***X*** and another element to ***β***.) The proper regression program gives estimates of the parameters, which we denote

$$\hat{\beta }=\left(\begin{array}{c} {\hat{\beta }}_{A}\\ {\hat{\beta }}_{B}\\ {\hat{\beta }}_{C}\\ \hat{\Delta } \end{array}\right).$$

Most programs will also produce an estimate of *σ*, which in this case has 6 degrees of freedom. If not, one can compute the residuals, which are given as the elements of the vector $y-X\hat{\beta }$, compute the sum of squares of the residuals, and divide the sum by the degrees of freedom to obtain an estimate of *σ*^2^. We denote this estimate by *s*^2^.

What we wish to investigate is the difference between indenters, *β*_A_ – *β*_B_, for example. We can easily estimate this difference as ${\hat{\beta }}_{A}-{\hat{\beta }}_{B}$ but obtaining a confidence interval is more involved. To express ${\hat{\beta }}_{A}-{\hat{\beta }}_{B}$ as the dot product of vectors, let

$$a_{\text{AB}}=\left(\begin{array}{c} 1\\ -1\\ 0\\ 0 \end{array}\right)$$

so that ${\hat{\beta }}_{A}-{\hat{\beta }}_{B}=a_{\text{AB}}^{T}\hat{\beta }$ As shown in texts on regression [8], an estimate of the covariance matrix of the parameter estimates is given by

$$s^{2}{\left(X^{T}X\right)}^{-1}.$$

Better regression programs give this matrix. Using this matrix, we obtain an estimate of the variance of the difference ${\hat{\beta }}_{A}-{\hat{\beta }}_{B}$ given by $s^{2}a_{\text{AB}}^{T}{\left(X^{T}X\right)}^{-1}a_{\text{AB}}$. Similarly, the variances of other differences can be obtained. Taking into account the 6 degrees of freedom in the variance estimate, we obtain the 95 percent confidence interval

$${\hat{\beta }}_{A}-{\hat{\beta }}_{B}\pm 2.447s\sqrt{a_{\text{AB}}^{T}{\left(X^{T}X\right)}^{-1}a_{\text{AB}}.}$$

**Fig. 7:**
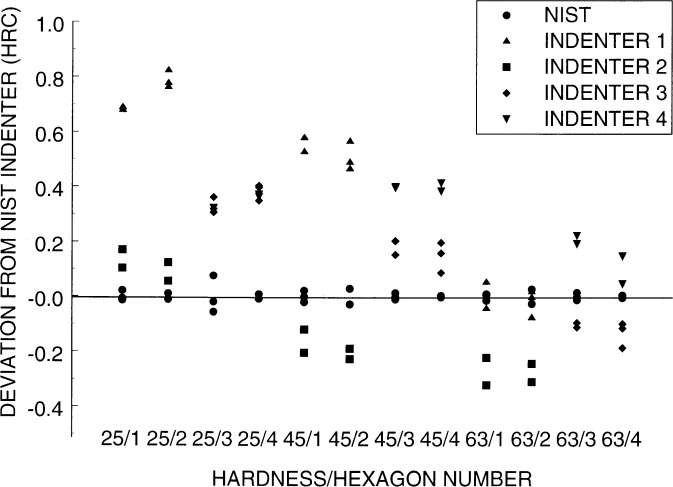
Measurements with five indenters given as deviations from the averages of measurements with the NIST indenter.

As an example, consider comparison of the NIST primary indenter with four other indenters. To accommodate this number of indenters, we implemented the above design twice on two sets of two hexagons. For both sets, the NIST indenter was assigned as the A indenter. Then, in the first set, indenters 1 and 2 were assigned to B and C, respectively, and in the second set, indenters 3 and 4 were assigned to B and C, respectively. The measurements are shown in Fig. 7 as deviations from the average of the measurements with the NIST indenter. Dominating Fig. 7 are the differences between the NIST indenter and the other four. We see, for example, that indenter 1 gives higher hardness readings at the 25 and 45 levels, and that indenter 2 gives lower readings at the 45 and 63 levels.

Figure 7 is affected by both block nonuniformity and lack of repeatability. The averages of cross hexagon pairs are not affected by block nonuniformity. The two hexagons in a set are nearly replicates. Thus, the results for all three indenters should line up. They do not because we centered them on the average of the measurements for the NIST indenter. The effect of the lack of repeatability on this centering can be taken into account by sliding the entire column of symbols up and down to achieve a better match. This is effective in some cases such as the rightmost two columns. Obtaining the best estimates of differences and an estimate of the standard deviation requires the regression methodology described above. We applied this methodology to each set of two hexagons and pooled the standard deviation estimates from the two sets. The results of this analysis in terms of differences from the NIST indenter are given in Table 1. The uncertainties are standard deviation estimates each with 12 degrees of freedom. One can use these to obtain confidence intervals.

### 3.7 More Elaborate Comparisons

Someone who is familiar with the matrix notation for multiple regression can generalize the foregoing to experimental designs involving different sets of comparisons or different numbers of hexagons. As an example, consider the following rigorous comparison of seven indenters labeled A through G. Generally, seven hexagons can be laid out on a block. One could assign the indenters to the across-hexagon pairs as shown in Table 2. One would also want to assign the center points of the hexagons. This plan, without the center points, is called a balanced incomplete block plan in the experimental design literature. In that literature, a block is a hexagon, not a test block. Analysis of data from such a design can be done through generalization of the multiple regression approach discussed in Sec. 3.6.

### 3.8 Reflections on Methodology

Constant hardness gradients across 6 mm hexagons only approximate block nonuniformity, although the approximation is useful as the methods in this section show. Referring to Fig. 3, one sees that hardness as a function of location is, at least in part, smoothly varying but this variation is only approximately planar across 6 mm hexagons. Thus, to some extent, block nonuniformity affects the results obtained with the methods in this section. The methods in Sec. 4 are based on a more realistic model. In this subsection, we consider the constant-gradient approximation in terms of the more realistic model used in the next section.

The model for block nonuniformity presented in Sec. 4 pictures the hardness variation as composed of two components, a smooth but curvilinear function and an irregularly varying function that appears to have no spatial continuity. The smooth component is reduced but not eliminated through the trend elimination methods presented in this section. The irregular component cannot be clearly distinguished from the lack of repeatability of the testing machine. Because a hardness measurement interferes with a subsequent measurement that is too close, one cannot tell whether what appears to be lack of repeatability is in part small-scale variation in the test block that does not appear smooth at permissible distances between measurements. In the use of the methods in this section, one can proceed as though what is observed is all due to lack of repeatability but realize that the resulting assessment of this uncertainty component may be too large. The model of block nonuniformity in the next section is actually a combined model of block nonuniformity and lack of repeatability.

In Sec. 4, we model block nonuniformity probabilistically. Such a model is supported by contour plots such as Fig. 3 obtained for other blocks. Comparing these contour plots shows that the hardness variation is largely smooth but shows little similarity from block to block. Thus, we treat the block nonuniformity as a largely smooth random function and estimate the covariance properties of this random function. In fact, the certificates that accompany NIST’s test blocks present such estimates [5]. If one had such estimates for one’s blocks, then one could estimate the part of the block nonuniformity that remains after trend elimination. Generally, of course, covariance properties are not available for the test blocks one is using and therefore, one would have to estimate them.

There are cases in which the irregular component in block nonuniformity might be distinguished from other error sources that influence the repeatability. Presumably, the irregular component has a constant variance for all blocks in a single manufacturing batch. Consider first the case of two machines. If, for a given batch of blocks, one machine has better repeatability than the other, then one can conclude that the extra variation observed in the poorer machine is due to error sources other than the irregular component of the block nonuniformity. Consider second the case of two batches of blocks. It seems possible that the same machine could exhibit different repeatability on two different batches of blocks. One could attribute such a difference to differences between the irregular components of the batches. One might ask whether different types of steel were used for the different batches, for example.

The methods in this section offer a solution to a problem in the use of several blocks in the same experiment. The problem arises when the measurement locations on a block are treated as randomly chosen. Even if such choice is properly made through designation of a set of locations on a block and random selection among these locations, the fact that the variance varies from block to block remains. The problem is that the variance induced by random selection depends on the nonuniformity of the block. The methods in this section remove the gross features of block nonuniformity and thus allow one to assume that whatever residual there is, it can be regarded as similarly distributed for every block in a manufacturing batch. Thus, the methods in this section allow pooling of variance estimates. Such pooling is also part of the estimation NIST uses in its block certification. Such pooling is especially valuable in the use of several blocks to apply control chart methodology over a long period.

## 4. Comparison with NIST

We now consider experimental methods based on the NIST SRMs. As shown in Sec. 3, there is much one can learn about a measurement system using any good-quality test blocks. More can be learned from NIST SRMs. In particular, judging the difference between what one’s system does and ideal execution of the Rockwell C Scale method is best done with NIST SRMs.

Test blocks, including NIST’s, are nonuniform. Think of the test surface of a NIST block as a collection of measurement locations. The metal at each of these locations will have a hardness value that differs slightly from the hardness values at other locations. If it were possible to determine the hardness at every location, then we would have hardness as a function of location. The function we would see would be largely smooth although perhaps also with a rapidly varying component. We expect the smooth part to be dominant because block nonuniformity is largely due to the nature of the steel and the test block manufacturing process, factors that vary smoothly across the block. NIST measured hardness as a function of location on several test blocks and found this to be true. Moreover, we found that these blocks have different hardness functions that are however, similar in their smoothness. Thus, a model that portrays each block as having a different but similarly smooth hardness function seems reasonable.

Generally, the user of a NIST SRM asks for the difference in hardness readings between the user’s own equipment and ideal equipment. The user might also ask for the difference between the user’s equipment and NIST’s. The day that NIST measures a user’s SRM block at the seven locations, it could also make measurements at many more locations. The user can ask what NIST would have obtained at other locations, in particular, at the locations of the user’s measurements. We now consider this question and answer it by providing a prediction of what NIST would have observed the same day as well as an uncertainty for this prediction. Note that this is not the same as a prediction of what NIST would have observed on a different day, which involves the NIST reproducibility. Also, note that in a comparison with user measurements, the user repeatability and reproducibility must be considered. We return to these uncertainty components in the next section. Because NIST could have continued to make measurements on an SRM block after it had made the initial seven, we can think in terms of a function of location ***s***, *H*(***s***), the value NIST would have observed at ***s*** the day it made the initial seven measurements.

The method we use to predict hardness values at untested locations is based on a geostatistics formula that models the hardness across the surface of a block as a random function described by a semivariogram [9]. The semivariogram is a mathematical model that describes how the measured hardness difference between any two test block locations relates to the physical spacing that separates them. In statistical terms, this semivariogram gives one half the variance of the hardness difference between any two locations on the test block. Thus, the square root of twice the semivariogram is the standard deviation of this difference.

We can obtain a semivariogram estimate from the measurements made on block 95N63001, which we have already depicted in Figs. 2 and 3. If we let *i* index these measurements, then for each value of *i* we have a hardness value *H_i_* and a location given by the coordinate values *x_i_* and *y_i_*. The semivariogram characterizes differences between these measurements as a function of the distance between them

$$d_{ij}=\sqrt{{\left(x_{i}-x_{j}\right)}^{2}+{\left(y_{i}-y_{j}\right)}^{2}.}$$

The semivariogram for a particular distance is estimated from the hardness differences for points that distance apart. Let $\hat{\gamma }\left(d\right)$ denote the estimated semivariogram for distance *d*. We have

$$2\hat{\gamma }\left(d\right)=\frac{1}{n_{d}}\sum_{d=d_{ij}}{\left(H_{i}-H_{j}\right)}^{2},$$

where the sum is over all the pairs of measurements (*i*, *j*) for which *d* = *d_ij_* and *n_d_* is the number of pairs in the sum. We see that the estimated semivariogram is one half the average of hardness differences squared and thus a variance estimate.

**Fig. 8:**
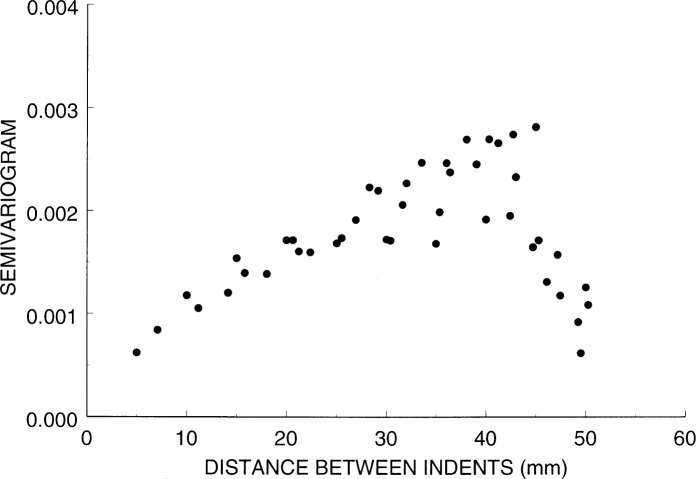
Empirical semivariogram for block 95N63001 (one half the mean square of differences).

A semivariogram characterizes the smoothness of the hardness measurements across a surface through the way it decreases with decreasing distance. The semivariogram estimate obtained from block 95N63001 is shown in Fig. 8. The important part of this semivariogram is the part for distances less than 26 mm, half the diameter of the block. The values for distances near 50 mm are estimated from the few pairs of points that span the block. That these values tend toward zero is the result of the concave hardness surface shown in Fig. 3, which is peculiar to this block. For distances less than 26 mm, the semivariogram decreases with decreasing distance as expected from the smoothness of the block nonuniformity. Note that the smallest distance is 5 mm, the smallest distance possible with a 5 mm grid. We would like to know the value that the semivariogram approaches as the distance approaches zero because this value characterizes the irregular component in the block nonuniformity and the lack of repeatability. One can estimate this value by extrapolating the values in Fig. 8 to zero. On this basis, Fig. 8 suggests that the repeatability of the NIST machine is very good. The value obtained by extrapolating to zero is, after taking the square root to convert it to a standard deviation, comparable to the repeatability results given in Sec. 3.3.

We estimated a semivariogram function for each SRM hardness range and use the result in our prediction of hardness values at unmeasured locations. This estimation is based on several blocks, not just one. An appropriate estimation algorithm is given by Curriero and Lele [10]. The algorithm actually used for the first batch of NIST Rockwell C scale SRMs is somewhat different but the resulting estimates are nearly the same. We fit an exponential semivariogram model to the data [9]. This model is given by

$$\gamma \left(d\right)=\{\begin{array}{cc} 0 & if\;d=0\\ c_{0}+c_{e}\left(1-\exp\left(-d/a_{e}\right)\right) & if\;d>0 \end{array}.$$

**Fig. 9:**
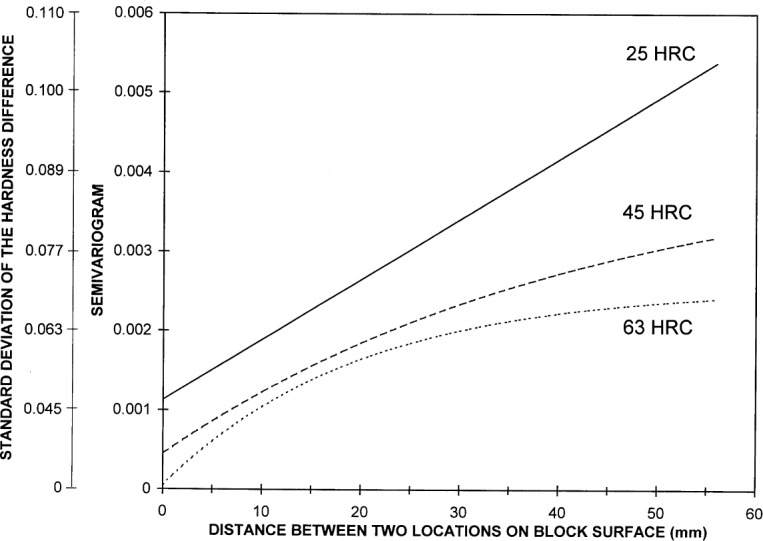
Semivariogram functions applicable to the first issue of the NIST SRMs.

The estimates are shown in Fig. 9. Note that these estimated functions provide an extrapolation to zero distance. Other extrapolations are possible. The way the zero value varies with hardness range raises an interesting question. Are there sources of error in NIST’s testing machine that are more severe for softer blocks or is there some variation in the blocks at distances much smaller than 5 mm that is more severe for softer blocks? Our data does not allow us to distinguish these alternatives.

Consider now prediction based on the seven NIST measurements, which are located at the vertices and at the center of a 20 mm hexagon. Let these initial locations be $s_{01},\;\ldots ,s_{0n_{0}}$ where *n*_0_ = 7. The values *H*(***s***_01_), …, *H*( $s_{0n_{0}}$) are provided on the SRM certificate. Consider another *n* points, ***s***_1_, …, ***s****_n_*. Of course, these *n* + 7 locations are subject to the minimum spacing requirements. We wish to predict

$$H_{\text{pred}}=\frac{1}{n}\sum_{k=1}^{n}H\left(s_{k}\right).$$

This formulation includes prediction for a single point (*n* = 1). We consider the more general case because users often make groups of measurements on test blocks. We compute not only a single-value prediction for this quantity, *Ĥ*_pred_, but also the prediction variance, $\sigma_{\text{pred}}^{2}$. This variance corresponds to the first source of uncertainty listed on the NIST certificate. Put together, we can obtain prediction intervals, for example, a 95 % interval (*Ĥ*_pred_ − 1.96*σ*_pred_, *Ĥ*_pred_ + 1.96*σ*_pred_).

The prediction *Ĥ*_pred_, which is a linear combination of the measurements on the certificate, is given by

$${\hat{H}}_{\text{pred}}=\sum_{i=1}^{n_{0}}\lambda_{i}H\left(s_{0i}\right),$$

where

$$\sum_{i=1}^{n_{0}}\lambda_{i}=1.$$

The predictions are based on the semivariograms given on the SRM certificates. For the first SRM batch, they are shown in Fig. 9. These semivariograms follow the exponential model given above and are functions of distance on the surface of the block. For convenience, we change our notation for the semivariogram. For two locations, ***s****_a_* and ***s****_b_*, separated by $\sqrt{{\left(x_{a}-x_{b}\right)}^{2}+{\left(y_{a}-y_{b}\right)}^{2}}$, we denote the value of the semivariogram by *γ*(***s****_a_* – ***s****_b_*) instead of by $\gamma \left(\sqrt{{\left(x_{a}-x_{b}\right)}^{2}+{\left(y_{a}-y_{b}\right)}^{2}}\right)$.

Computation of the coefficients and the prediction variance consists of four steps. The first step is inversion of the *n*_0_ × *n*_0_ matrix *Γ* with *i*, *j* element γ(***s***_0_*_i_* – ***s***_0_*_j_*). Let the elements of the inverse *Γ*^−1^ be denoted *g_ij_*. This inverse matrix depends only on the semivariogram for the points measured by NIST and therefore can be computed once for each hardness level. The second step is computation of

$${\bar{\gamma }}_{i}=\frac{1}{n}\sum_{k=1}^{n}\gamma \left(s_{k}-s_{0i}\right).$$

The third step is computation of three quadratic forms

$$\begin{array}{l} Q_{22}=\sum_{i=1}^{n_{0}}\sum_{j=1}^{n_{0}}{\bar{y}}_{i}\;g_{ij}{\bar{y}}_{j}\\ Q_{12}=\sum_{i=1}^{n_{0}}\sum_{j=1}^{n_{0}}\;g_{ij}{\bar{y}}_{j}\\ Q_{11}=\sum_{i=1}^{n_{0}}\sum_{j=1}^{n_{0}}\;g_{ij}. \end{array}$$

The final step is computation of the coefficients that multiply the NIST measurements *H*(***s***_0_*_i_*) to form the prediction *Ĥ_pred_* and the prediction variance

$$\begin{array}{l} \lambda_{i}=\sum_{j=1}^{n_{0}}g_{ij}\;{\bar{\gamma }}_{j}+\frac{\left(1-Q_{12}\right)}{Q_{11}}\sum_{j=1}^{n_{0}}g_{ij}\\ \sigma_{\text{pred}}^{2}=Q_{22}-\frac{{\left(Q_{12}-1\right)}^{2}}{Q_{11}}-\frac{1}{n^{2}}\sum_{k=1}^{n}\sum_{k^{\prime }=1}^{n}\gamma \left(s_{k}-s_{k^{\prime }}\right) \end{array}$$

Put together, these two quantities give a prediction interval for the average value NIST would have obtained on the day the SRM measurements were obtained. For example, a 95 % interval is given by

$${\hat{H}}_{\text{pred}}\pm 1.96\sigma_{\text{pred}}=\sum_{i=1}^{n_{0}}\lambda_{i}\;H\left(s_{0i}\right)\pm 1.96\sigma_{\text{pred}}.$$

**Fig. 10:**
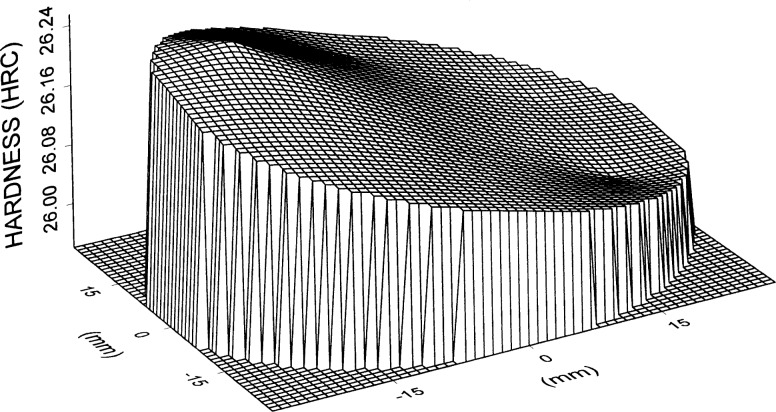
Hardness values of unmeasured locations on a NIST SRM block predicted from the seven measurements NIST made.

An example of prediction for *n* = 1 is shown in Fig. 10. This figure shows the predicted hardness *Ĥ*_pred_ for each location on a block, actually a low-range block, based on the seven measurements made on this block. We see that in terms of gross features, the nonuniformity of this block differs from the nonuniformity of block 95N63001 shown in Fig. 3.

The foregoing computational details provide little insight into the prediction itself. As a consequence of the semivariogram estimates and the locations of the NIST measurements on a 20 mm hexagon, the values of λ*_i_* that result from the above computation are positive (or at least close to being positive). This implies that the predicted value must lie between the smallest and the largest of the NIST measurements. In the case *n* = 1, *Ĥ*_pred_ can be regarded as an interpolated value. In fact, if only two points were measured (*n*_0_ = 2) and the prediction were for the point half way between them, then the prediction would be the average of the two measurements.

For the purpose of correcting for machine and indenter error as discussed in Sec. 5 and for other metrological purposes, it is best to use *Ĥ*_pred_. Current practice, however, is to provide a single hardness value for a test block. On its certificates, NIST also provides a single value for a block. This certified average hardness value is an estimate of the hardness function integrated over the test block surface divided by the surface area. It is analogous to the certified value assigned to commercially produced hardness test blocks, which is usually calculated as the arithmetical average of the measurements. In the case of the NIST block, the certified average hardness value is the average of the predicted hardness values for all test surface locations, and not the arithmetical average of the seven NIST measurements. Because the locations chosen for the seven NIST measurements provide a good representation of the range in surface hardness, the two averages are nearly identical in value.

Some blocks such as the one portrayed in Fig. 3, we measured more than just the seven times that is characteristic of the SRM blocks NIST offers. For these blocks, we can choose *n*_0_ measurements, use these *n*_0_ measurements to predict the other measurements, and compare the prediction *Ĥ*_pred_ with the actualization *H*_pred_. The question of how well the prediction performs has two aspects. One is whether the prediction interval contains the actualization with the frequency implied by the percent confidence chosen. The other is whether subtracting the prediction from the actualization reduces the variation by a substantial amount. There are some details to be considered in this performance investigation. We used two sets of locations in filling blocks with measurements. This results in what we refer to as filled blocks and partially filled blocks. Unfortunately, neither set of locations contains the SRM locations. For this reason, we chose other locations as the basis for the prediction.

**Fig. 11:**
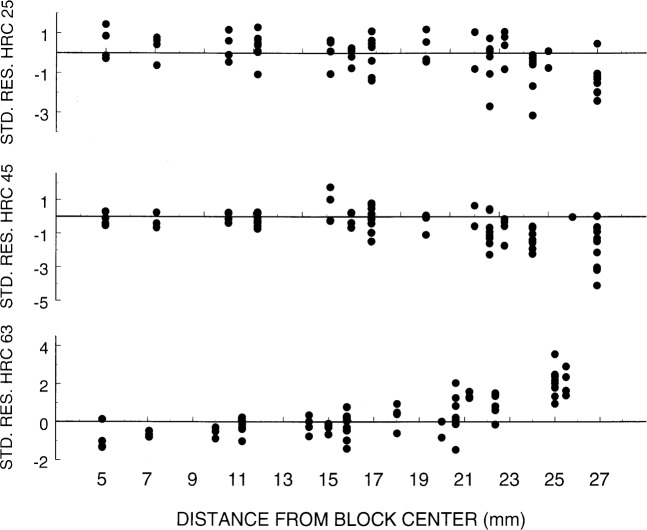
Measured value minus predicted value divided by standard deviation of the predicted value for a block of each hardness level.

We consider the first aspect using a filled block for each hardness range. Because the locations actually measured on the SRM blocks were not measured on the filled blocks, we use as a basis for prediction the measurements at the points (0,0), (−20,0), (−10,15), (10,15), (20,0), (10, −15) and (−10, −15). For each location not in this set, we computed the predicted value, subtracted it from the actualization, and divided by the prediction standard deviation to obtain (*H*_pred_ – *Ĥ*_pred_)/*σ*_pred_, where *n* = 1. Note that the actualization is the value we set out to predict, which is the value NIST obtained. We call these values standardized residuals and show them in Fig. 11.

The values shown in this figure should ideally lie outside (−1.96, 1.96) only one time in twenty. Since we have plotted the values versus the distance of the location from the center of the block, we can see that the statistical model on which the prediction interval is based does not hold exactly. We see an edge effect that is not surprising when one thinks of how test blocks are manufactured. On these blocks, the hardness near the edge varies more rapidly with distance than is portrayed by the semivariogram. This is particularly true of the HRC 63 block. The contour for this block is shown in Fig. 3. It is not clear what can be done about this edge effect. First, not all blocks have edge effects, and in fact, the contours for different blocks show little resemblance. Thus, use of a model of the block nonuniformity that takes into account the edges does not seem worthwhile. Second, in finding the difference between the user’s equipment and NIST’s, the user will make more than one measurement, and this will relieve the problem unless the user makes all the measurements near the edge. We conclude that if the user is somewhat cautious about measurements near the edge of the block, the prediction approach presented here is serviceable.

We investigate the second aspect of prediction performance using three partially filled blocks. We compare the variation of the actualization with the variation of the difference between the actualization and the prediction. Because of the locations measured on the partially filled blocks, we cannot predict on the basis of a hexagon pattern of points. We used instead the six points (−22, 0), (−5, 0), (5, 0), (23, 0), (0, −15), and (0, 15), a cross pattern. We predicted the other points on the block for which we already had hardness measurements, the actualizations. Let *S*_1_ be the centered sum of squares of the actualizations *H*_pred_ for the locations predicted, and let *S*_2_ be the centered sum of squares of the corresponding residual values *H*_pred_ – *Ĥ*_pred_. The difference *S*_1_ – *S*_2_ shows how well the prediction corresponds to the actualization. We compute

$$R^{2}=\left(S_{1}-S_{2}\right)/S_{1}.$$

For the low range, mid range, and high range, we obtain for *R*^2^ the values 0.69, 0.61, and 0.39. In the interpretation of these values, note first that *R*^2^ behaves like the similar quantity in regression analysis; the value 1 corresponds to perfect prediction. Moreover, the value of *R*^2^ will generally be higher for blocks that are more nonuniform. The values we obtained from the blocks considered are evidence of how large *R*^2^ might be amidst the first batch of NIST SRMs. The values for more uniform blocks would be smaller. What the effect of substituting the hexagon pattern for the cross pattern would be, we do not know. Nevertheless, we see that for the more nonuniform blocks in a batch, prediction reduces the variation substantially.

## 5. Measurement Correction

### 5.1 User-NIST Difference

People who make hardness measurements should expect their results to differ from results NIST would have obtained with its indenter and testing machine and, for this reason, should entertain correction of their measurements so that the agreement is better. The procedures in this section are a guide to making such corrections and, in addition, a guide to deciding whether such corrections are satisfactory. If they are unsatisfactory, equipment upgrades may be the only option.

Users of NIST’s Rockwell C scale SRMs can observe the difference between their measurements and NIST’s at the three hardness levels for which SRMs are offered. More precisely, as discussed in Sec. 4, users can observe the difference between the average of their measurements on an SRM block and a prediction of what NIST would have obtained for the same locations. This section shows how a correction of a user measurement at any hardness level can be obtained from the differences at the three available hardness levels. Correction, however, may not provide sufficiently small measurement uncertainty. In this section, we discuss measurement correction including all the uncertainty components that a corrected measurement entails. After computing the total uncertainty from the components, the user can decide whether measurement correction is sufficient or whether an equipment upgrade is needed.

In Sec. 3.5, we expressed a hardness measurement as the sum of three terms, *H* = *µ* + *δ* + *ε*, where *µ* involves the actual hardness as well as the machine and indenter errors. In the case of the user machine and indenter, we use the symbol *µ*. In the case of the NIST machine and indenter, we use the symbol *µ*_NIST_. Ideally, one would like to correct *µ* to the actual hardness value. However, because the NIST SRMs involve machine and indenter error, it is better to think of correcting *µ* to *µ*_NIST_. The correction is

$$f\left(\mu_{\text{NIST}}\right)=\mu -\mu_{\text{NIST}},$$

and therefore, we have

$$H=\mu_{\text{NIST}}+f\left(\mu_{\text{NIST}}\right)+\delta +\varepsilon .$$

On this basis, we correct measurements to the scale defined by the NIST machine and indenter.

The correction is determined from user measurements on a NIST block for each hardness level. The first question is choice of measurement locations. Actually, one can make measurements on a NIST SRM wherever one wants (subject to the minimum spacing requirements) and use the equations in Sec. 4 to obtain the NIST certified value (with uncertainty) for the average of these locations. Nevertheless, some choices of locations are better than others. Although we have not studied this issue, a reasonable possibility seems to be an extension of the 6 mm hexagons discussed above. One can lay out 6 mm hexagons around each of the NIST measurements and select one or more cross-hexagon pairs as one’s measurement locations. In comparing an across-hexagon average with the center value, one should not assume that the block nonuniformity cancels as we do in Sec. 3. The prediction uncertainty produced by the equations in Sec. 4 takes into account the fact that the nonuniformity does not cancel exactly. Thus, even though one chooses locations based on 6 mm hexagons, one should use Sec. 4 to obtain the NIST prediction interval.

Say that one has made *n* measurements on a NIST SRM and that the average of these measurements is $\bar{H}$. Moreover, say that one has followed Sec. 4 to obtain the NIST prediction for the average hardness of these locations *Ĥ*_pred_. The difference $\bar{H}$ − *Ĥ*_pred_ would not be zero even if $\bar{H}$ had been obtained with the same machine and indenter NIST used to certify its SRMs. Using bars to denote averages, we obtain from the equation for *H* given above

$$\bar{H}=\mu_{NIST}+f\left(\mu_{NIST}\right)+\delta +\bar{\varepsilon }.$$

The standard deviation of *δ* is *σ_δ_*, and the standard deviation of $\bar{\varepsilon }$ is $\sigma /\sqrt{n}$. Moreover, think of *Ĥ*_pred_ as composed of three components

$${\hat{H}}_{\text{pred}}=\mu_{\text{NIST}}+\delta_{\text{NIST}}+\varepsilon_{\text{pred}},$$

where *µ*_NIST_ is the actual hardness reading corrupted by the NIST machine and indenter error, *δ*_NIST_ reflects NIST’s lack of reproducibility, and *ε*_pred_ is the prediction error discussed in Sec. 4. The standard deviation of *δ*_NIST_, which we denote _−NIST_, is given on the *σ_δ_*_−NIST_ certificate. We have

$$\bar{H}-{\hat{H}}_{\text{pred}}=f\left(\mu_{\text{NIST}}\right)+\delta +\bar{\varepsilon }-\delta_{\text{NIST}}-\varepsilon_{\text{pred}}.$$

The combined standard deviation for the last four terms in this equation is

$$\sigma_{\Delta }=\sqrt{\sigma_{\delta }^{2}+\sigma^{2}/n+\sigma_{\delta -\text{NIST}}^{2}+\sigma_{\text{pred}}^{2}}.$$

Estimation of *σ*^2^ is discussed in Sec. 3.3; estimation of $\sigma_{\delta }^{2}$ is discussed in Sec. 3.5; the value of $\sigma_{\delta -\text{NIST}}^{2}$ can be obtained from the certificate; and computation of $\sigma_{\text{pred}}^{2}$ is discussed in Sec. 4.

Comparison of one’s hardness system with NIST’s requires consideration of all three hardness levels for which NIST has issued SRMs. Let *m* index these hardness levels. We write ${\bar{H}}_{m}-{\hat{H}}_{\text{pred}\left(m\right)},\;\mu_{m}-\mu_{\text{NIST}\left(m\right)}$ and *σ*_Δ_*_m_*. The random errors *δ*, $\bar{\varepsilon }$, *δ*_NIST_, *ε*_pred_ in the expression for ${\bar{H}}_{m}-{\hat{H}}_{\text{pred}}\left(m\right)$ occur at each hardness level and thus should have the subscript *m*. Three of these random errors, $\bar{\varepsilon }$, *δ*_NIST_, *ε*_pred_, are statistically independent from one hardness level to another. The fourth, *δ*, the error associated with the user reproducibility, may not be statistically independent from one hardness level to another, but we assume that it is.

### 5.2 Curvature

Two aspects of *f*(*µ*NIST) distinguish hardness measurement correction from calibration problems in other fields. First, over the range of Rockwell C scale hardness, the function *f*(*µ*NIST) is smooth and small. This will be true if the user’s machine and indenter are reasonably close to the Rockwell C scale prescription. On this basis, we can replace *f*(*µ*_NIST_) with *f*(*Ĥ*_pred_). Second, the function *f* exhibits some curvature. For this reason, we must include deviations of *f*(*µ*_NIST_) from linearity as an uncertainty component.

We approximate *f*(*µ*_NIST_) by *a* + (*β* – 1) *µ*_NIST_. Because the NIST blocks have hardness values near 25, 45, and 65 HRC, we can check this approximation by considering the difference between the deviation for the middle block and the average of the deviations for the highest and lowest blocks. Let

$$r_{3}=\frac{{\hat{H}}_{\text{pred}\left(2\right)}-{\hat{H}}_{\text{pred}\left(1\right)}}{{\hat{H}}_{\text{pred}\left(3\right)}-{\hat{H}}_{\text{pred}\left(1\right)}}$$

and let

$$r_{1}=\frac{{\hat{H}}_{\text{pred}\left(3\right)}-{\hat{H}}_{\text{pred}\left(2\right)}}{{\hat{H}}_{\text{pred}\left(3\right)}-{\hat{H}}_{\text{pred}\left(1\right)}}$$

If we ignore the error in these ratios, then this gauge of curvature is given by

$$\theta =r_{3}\left(\mu_{3}-\mu_{\text{NIST}\left(3\right)}\right)+r_{1}\left(\mu_{1}-\mu_{\text{NIST}\left(1\right)}\right)-\left(\mu_{2}-\mu_{\text{NIST}\left(2\right)}\right)$$

and is estimated by

$$\hat{\theta }=r_{3}\left({\bar{H}}_{3}-{\hat{H}}_{\text{pred}\left(3\right)}\right)+r_{1}\left({\bar{H}}_{1}-{\hat{H}}_{\text{pred}\left(1\right)}\right)-\left({\bar{H}}_{2}-{\hat{H}}_{\text{pred}\left(2\right)}\right)$$

The standard deviation of $\hat{\theta }$ is given by

$$\sqrt{r_{3}^{2}\sigma_{\Delta 3}^{2}+r_{1}^{2}\sigma_{\Delta 1}^{2}+\sigma_{\Delta 2}^{2}}$$

Using this standard deviation, one can form a confidence interval for *θ* and judge how large *θ* might be in light of the various sources of random error. If *θ* is small in reference to the measurement application, then a linear measurement correction is reasonable. Because NIST issues blocks at only three hardness levels, we consider only the possibility of a correction linear in the user’s reading since there would be no way to check the fit of a curvilinear correction.

### 5.3 Linear Correction

We now consider estimating the correction and the uncertainty associated with this estimation. First, we calculate the average of the NIST values

$${\hat{H}}_{\text{avg}}=\frac{1}{3}\sum_{m=1}^{3}{\hat{H}}_{\text{pred}\left(m\right)},$$

the estimated slope

$$\hat{\beta }-1=\frac{\sum_{m=1}^{3}\left({\bar{H}}_{m}-{\hat{H}}_{\text{pred}\left(m\right)}\right)\left({\hat{H}}_{\text{pred}\left(m\right)}-{\hat{H}}_{\text{avg}}\right)}{\sum_{m=1}^{3}{\left({\hat{H}}_{\text{pred}\left(m\right)}-{\hat{H}}_{\text{avg}}\right)}^{2}}$$

and the estimated intercept

$$\hat{\alpha }=\frac{1}{3}\sum_{m=1}^{3}\left({\bar{H}}_{m}-{\hat{H}}_{\text{pred}\left(m\right)}\right)-\left(\hat{\beta }-1\right){\hat{H}}_{\text{avg}}.$$

Denote the hardness reading to be corrected as *U*. The correction to be subtracted from *U* is

$$C=\left[\hat{\alpha }+\left(\hat{\beta }-1\right)U\right]/\hat{\beta }.$$

Thus, the corrected reading is *U* – *C*. In terms of the standard deviation, the uncertainty in *C* is given by

$$\sigma_{C}={\left[\sum_{m=1}^{3}{\left(\frac{1}{3}+\frac{\left(U-{\hat{H}}_{\text{avg}}\right)\left({\hat{H}}_{\text{pred}\left(m\right)}-{\hat{H}}_{\text{avg}}\right)}{\Sigma_{m=1}^{3}{\left({\hat{H}}_{\text{pred}\left(m\right)}-{\hat{H}}_{\text{avg}}\right)}^{2}}\right)}^{2}\left(\sigma_{\Delta m}^{2}\right)\right]}^{1/2}.$$

### 5.4 Uncertainty

Consider finally, the uncertainty components of *U* – *C*, a user measurement corrected to the NIST scale. To begin, one must ask about the scale to which the uncertainty refers. Since the measurement is corrected to the NIST scale, it is reasonable to take the NIST scale as the truth to which the uncertainty refers. In this case, there are four uncertainty components, the one due to user lack of repeatability, the one due to user lack of reproducibility, the one due to error in estimation of the linear correction, and one that must account for the curvature in the relation between user measurements and NIST’s. In terms of the usual uncertainty parlance, the first three of these are Type A [4]. The standard uncertainties of these components are given above. The uncertainty that arises because the correction really needed is non-linear can be gauged by *θ*. From the confidence interval for *θ*, we can develop bounds on the uncertainty due to this source. Such bounds should be serviceable if not completely defensible because *f*(*µ*_NIST_) could take an even greater excursion. With these uncertainty components assessed, one is in a position to decide whether one’s measurements after correction are sufficient for the purpose.

The implications of $\hat{\theta }$ can be further understood in terms of its relation to $\hat{\alpha }$ and $\hat{\beta }$. The estimates $\hat{\theta }$, $\hat{\alpha }$, and $\hat{\beta }$ provide an approximate decomposition of ${\bar{H}}_{m}-{\hat{H}}_{\text{pred}\left(m\right)}$, *i* = 1, 2, 3. This approximation is exact if *Ĥ*_pred(1)_ = 25, *Ĥ*_pred(2)_ = 45, and *Ĥ*_pred(3)_ = 65. We consider this special case because the NIST SRMs are approximately at these levels. With some algebra, we see that

$$\begin{array}{l} \hat{\theta }=\left({\bar{H}}_{1}+{\bar{H}}_{3}\right)/2-{\bar{H}}_{2}\\ \hat{\alpha }=\left({\bar{H}}_{1}+{\bar{H}}_{2}+{\bar{H}}_{3}\right)/3-45\hat{\beta }\\ \hat{\beta }=\left({\bar{H}}_{3}-{\bar{H}}_{1}\right)/40 \end{array}$$

The decomposition is

$$\begin{array}{l} {\bar{H}}_{1}-25=\hat{\alpha }+25\left(\hat{\beta }-1\right)+\hat{\theta }/3\\ {\bar{H}}_{2}-45=\hat{\alpha }+45\left(\hat{\beta }-1\right)-2\hat{\theta }/3\\ {\bar{H}}_{3}-65=\hat{\alpha }+65\left(\hat{\beta }-1\right)+\hat{\theta }/3 \end{array}$$

We see that the user values are decomposed into a linear function of the NIST predictions *Ĥ*_pred(_*_m_*_)_ and our gauge of curvature $\hat{\theta }$. Thus, $\hat{\theta }$ accounts for the part of the user measurements not amenable to a linear correction. Moreover, if $\sigma_{\Delta m}^{2}$ is the same for all *m*, then the estimation error for $\hat{\alpha }+{\hat{H}}_{\text{pred}\left(m\right)}\left(\hat{\beta }-1\right)$ is independent of the estimation error for $\hat{\theta }$. Thus, the uncertainty in the linear correction and the uncertainty in the gauge of curvature $\hat{\theta }$ when combined as the root sum of squares gives approximately the uncertainty in ${\bar{H}}_{m}-{\hat{H}}_{\text{pred}\left(m\right)}$. These results show that because of the levels of the NIST SRMs, the quantities introduced in this section are more simply related than might appear at first. Of particular note is the fact that applying the linear correction does not increase the uncertainty in user measurements.

A completely defensible recipe for incorporating the curvature into the uncertainty does not seem possible. Our gauge of curvature, a confidence interval for *θ*, does not tell us about the differences between user and NIST measurements at hardness levels between the levels of the NIST SRMs. These differences could be larger than 2*θ*/3. Moreover, these differences could be material dependent. Such dependence might be especially severe when *θ* is large. To this must be added the complication that, as shown above, the effect of the curvature on the corrected user measurements is $\hat{\theta }/3$ or $-2\hat{\theta }/3$ depending on the level of the NIST SRM. A consensus recipe for the case of *θ* small may be possible, but if the confidence interval for *θ* suggests that *θ* may be large, the user of the uncertainty statement must be careful.

Unless all parties agree to correct their measurements to the NIST scale, one must consider the relation of measurements to the ideal Rockwell C scale. One way to do this is to add to the above uncertainty components, the uncertainty components given on the NIST certificate for machine and indenter error. This will increase the total uncertainty. The advantage of agreement to correct to the NIST scale is that the NIST machine and indenter errors do not have to be considered in a comparison. Of course, it would be still better to improve machines and indenters so that errors attributable to these sources are reduced.

## 6. Summary

Rockwell hardness occupies a preeminent position in mechanical testing because of its long history and wide use. For this reason, Rockwell hardness is the natural choice for a paper on detailed understanding of test methods. This paper provides procedures that can be implemented to evaluate a hardness measurement system, concepts that help with the investigation of hardness measurement, and an example that can guide test method development.

This paper provides procedures for repeatability and reproducibility assessment, for control charts, for comparison of systems, for use of NIST SRM test blocks, and for measurement correction. These procedures give a fine understanding, but a reader may ask whether such an understanding is necessary. The answer is, of course, that it depends on the hardness application.

This paper explains concepts related to sources of measurement uncertainty, use of nonuniform blocks, the combined uncertainty of measurement, and evidence of the need for equipment upgrades. For a reader involved in hardness testing, these concepts are important even if the procedures in this paper are not implemented. For example, if one is puzzling over why two hardness measurements differ, one needs these concepts in thinking about possible causes.

Although it does not apply to all test methods, this paper does apply to many tests currently under development. Generally, this paper applies to tests for products with both upper and lower specification limits. One general aspect of test method development discussed in this paper is the need to manage two distinct areas of experimental effort, assessment of measurement uncertainty and determination of the relation between the test method and the product property critical to quality. A second aspect of broad interest is the use of test methods for local measurements of surfaces. Applicable to such a test method use are approaches to surface variation such as trend elimination and spatial statistics. A third aspect is the need for reference materials to assure long-term comparability of test method results. In general, this paper shows how test methods can be treated with more care than is usual in current practice.

## Figures and Tables

**Table 1:** Differences from NIST indenter of indenters 1 to 4 each given with 1 standard uncertainty and the estimated standard deviation of the lack of repeatability

	HRC 25	HRC 45	HRC 63
Indenter 1	0.742±0.021	0.532±0.016	−0.008±0.018
Indenter 2	0.111±0.022	−0.182±0.017	−0.274±0.019
Indenter 3	0.357±0.021	0.161±0.016	−0.113±0.018
Indenter 4	0.338±0.022	0.399±0.017	0.156±0.019
Std. Dev. *s*	0.033	0.025	0.028

**Table 2:** Design for comparison of indenters A to G

Hexagon	1, 4 pair	2, 5 pair	3, 6 pair
1	A	B	D
2	B	C	E
3	C	D	F
4	D	E	G
5	E	F	A
6	F	G	B
7	G	A	C
